# Alternative Splicing Regulator RBM20 and Cardiomyopathy

**DOI:** 10.3389/fmolb.2018.00105

**Published:** 2018-11-28

**Authors:** Takeshi Watanabe, Akinori Kimura, Hidehito Kuroyanagi

**Affiliations:** ^1^Laboratory of Gene Expression, Medical Research Institute, Tokyo Medical and Dental University (TMDU), Tokyo, Japan; ^2^Department of Psychosomatic Dentistry, Graduate School of Medical and Dental Science, Tokyo Medical and Dental University (TMDU), Tokyo, Japan; ^3^Division of Pathology, Department of Molecular Pathogenesis, Medical Research Institute, Tokyo Medical and Dental University (TMDU), Tokyo, Japan; ^4^Laboratory for Integrated Research Projects on Intractable Diseases Advanced Technology Laboratories, Medical Research Institute, Tokyo Medical and Dental University (TMDU), Tokyo, Japan; ^5^Department of Microbiology, Immunology and Molecular Genetics, University of California, Los Angeles, Los Angeles, CA, United States

**Keywords:** RBM20, dilated cardiomyopathy (DCM), alternative splicing, isoform switching, mutation, arginine/serine (RS)-rich region, titin, nuclear localization

## Abstract

RBM20 is a vertebrate-specific RNA-binding protein with two zinc finger (ZnF) domains, one RNA-recognition motif (RRM)-type RNA-binding domain and an arginine/serine (RS)-rich region. *RBM20* has initially been identified as one of dilated cardiomyopathy (DCM)-linked genes. RBM20 is a regulator of heart-specific alternative splicing and *Rbm20*^Δ*RRM*^ mice lacking the RRM domain are defective in the splicing regulation. The *Rbm20*^Δ*RRM*^ mice, however, do not exhibit a characteristic DCM-like phenotype such as dilatation of left ventricles or systolic dysfunction. Considering that most of the *RBM20* mutations identified in familial DCM cases were heterozygous missense mutations in an arginine-serine-arginine-serine-proline (RSRSP) stretch whose phosphorylation is crucial for nuclear localization of RBM20, characterization of a knock-in animal model is awaited. One of the major targets for RBM20 is the *TTN* gene, which is comprised of the largest number of exons in mammals. Alternative splicing of the *TTN* gene is exceptionally complicated and RBM20 represses >160 of its consecutive exons, yet detailed mechanisms for such extraordinary regulation are to be elucidated. The *TTN* gene encodes the largest known protein titin, a multi-functional sarcomeric structural protein specific to striated muscles. As titin is the most important factor for passive tension of cardiomyocytes, extensive heart-specific and developmentally regulated alternative splicing of the *TTN* pre-mRNA by RBM20 plays a critical role in passive stiffness and diastolic function of the heart. In disease models with diastolic dysfunctions, the phenotypes were rescued by increasing titin compliance through manipulation of the *Ttn* pre-mRNA splicing, raising RBM20 as a potential therapeutic target.

## Introduction

Cardiomyopathy is a myocardial disease with cardiac dysfunction. Cardiomyopathy is roughly classified as genetic cardiomyopathy including hypertrophic cardiomyopathy (HCM), and mixed (genetic and acquired) cardiomyopathy such as dilated cardiomyopathy (DCM; Dadson et al., 2017). HCM is a disease in which hypertrophy of the ventricle occurs despite the absence of high blood pressure or valvular disease that cause ventricular hypertrophy (Elliott, 2014). More than half of the HCM patients carry mutations in one of eight sarcomere genes (Sabater-Molina et al., 2018). DCM is another common form of cardiomyopathy, affecting ~1 in 250–500 in general population (McKenna et al., 2017) and characterized by left ventricular dilatation and systolic dysfunction in the absence of abnormal loading conditions or coronary artery disease (Rampersaud et al., 2011; McCartan et al., 2012). Mortality rate of DCM is high as a result of heart failure (Kirk et al., 2009). Among the idiopathic DCM cases, 20–35% are familial, with autosomal dominant inheritance in most cases (Kimura, 2016). A next generation sequencing method has recently identified more than 400 potentially causative mutations in 60 genes both in familial and sporadic DCM cases (Pérez-Serra et al., 2016). These DCM-associated genes can be classified into various functional groups such as muscle contraction, Ca^2+^ handling, and nuclear function. Such molecular genetic complexity makes it difficult to elucidate the mechanisms bringing about the common phenotypes of DCM (Hershberger et al., 2013). Among the DCM-linked mutations, 25% were mapped to the *TTN* gene (Herman et al., 2012; Hershberger et al., 2013; Fatkin and Huttner, 2017). The human *TTN* gene has 364 exons, the largest number of exons in a single gene in mammals, 363 of which are coding. The *TTN* gene encodes the largest known protein titin, a multi-functional sarcomeric structural protein specific to striated muscles (Gigli et al., 2016). Titin plays a major role in passive tension of cardiomyocytes (Hidalgo and Granzier, 2013). The *TTN* pre-mRNA undergoes extensive alternative splicing, leading to tissue-specific and developmentally regulated titin isoforms.

*RBM20*, encoding RNA Binding Motif Protein-20 (RBM20) has initially been identified as one of the DCM-linked genes (Brauch et al., 2009). Genetic abnormalities in *RBM20* have been identified in about 2–3% of familial and sporadic DCM cases (Li et al., 2010; Refaat et al., 2012; Kayvanpour et al., 2017). Recently, RBM20 has been identified as a crucial RNA-binding protein that controls the splicing of *TTN* (Guo et al., 2012). However, roles of RBM20 in the pathophysiology of DCM is still unclear. Only a few studies have addressed molecular mechanisms of splicing regulation by RBM20, and are controversial. In this review, we summarize the literature on RBM20 and discuss effects of mutations found in the DCM patients. We also summarize recent attempts to manipulate the RBM20 functions in various disease models. Finally, we will discuss open questions about the functions of RBM20 and its relevance to DCM.

## Structure of RBM20

**Figure 1:**
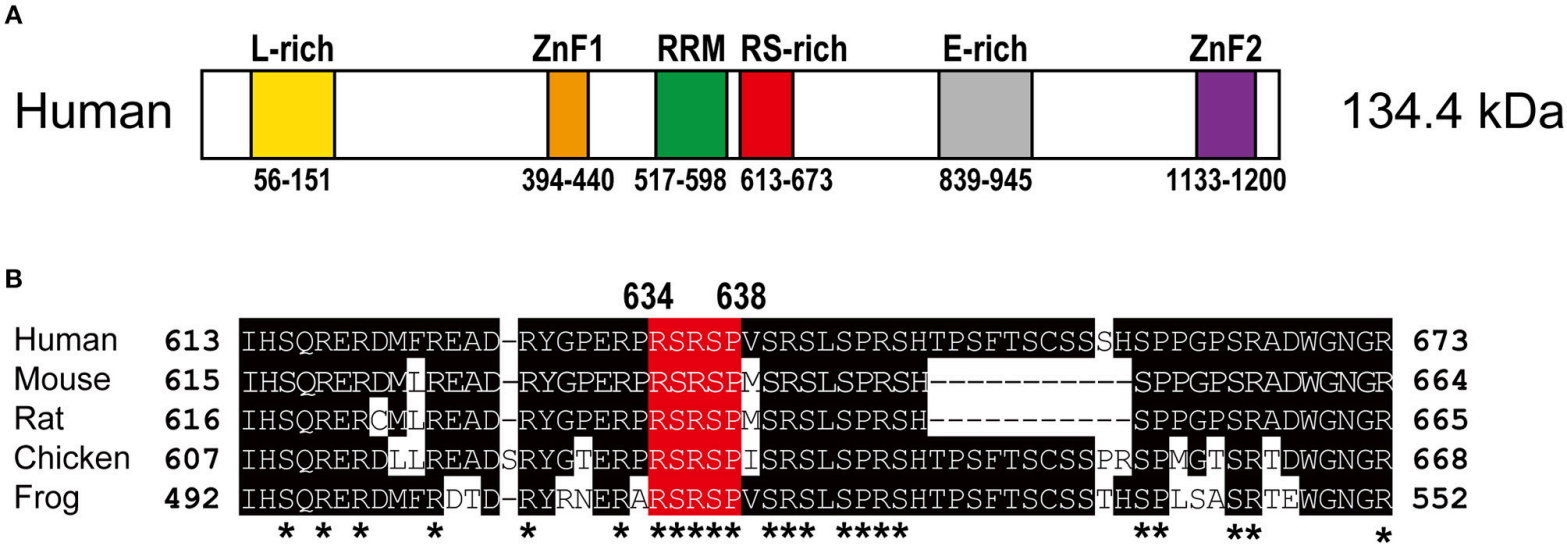
Structure of the RBM20 protein. **(A)** Schematic domain structure of the human RBM20 protein. Names and positions of the domains are indicated. E-rich, glutamate-rich region; L-rich, leucine-rich region; RRM, RNA-recognition motif domain; RS-rich, arginine/serine-rich region; ZnF, zinc finger domains. **(B)** Amino acid sequence alignment of the RS-rich region of RBM20 proteins from human, mouse, rat, chicken and frog. Amino acid residues that match the human RBM20 residues are shaded. The RSRSP stretch is in red. Asterisks indicate evolutionarily conserved arginine (R), serine (S), and proline (P) residues.

RBM20 is a vertebrate-specific RNA-binding protein (Zerbino et al., 2018). The human *RBM20* gene resides on chromosome 10 and is composed of 14 exons. Human RBM20 protein consists of 1,227 amino acid residues and is relatively large for a splicing regulator, yet it has only three conserved recognizable functional domains: two zinc finger (ZnF) domains and one RNA-Recognition Motif (RRM)-type RNA-binding domain (Figure 1). Sequence alignment of RBM20 proteins from various vertebrate species revealed three other conserved regions (Guo et al., 2012; Murayama et al., 2018; Zahr and Jaalouk, 2018): a leucine (L)-rich region at the N-terminus, an arginine/serine (RS)-rich region just downstream from the RRM domain and a glutamate (E)-rich region between the RS-rich region and the ZnF2 domain (Figure 1).

Vertebrates have two proteins homologous to RBM20; matrin3, and ZNF638 have two RRM domains sandwiched by two ZnF domains and these domains are most related to those of RBM20 (Coelho et al., 2016). RBM20 is highly expressed in the heart and the skeletal muscle (Filippello et al., 2013), whereas matrin3 and ZNF638 are widely expressed across different cell types (Coelho et al., 2016).

## *RBM20* mutations in DCM patients

The *RBM20* mutations identified so far in familial as well as sporadic DCM cases are listed in Table 1. The list clearly revealed that almost all of the *RBM20* mutations are heterozygous missense mutations and are enriched in a hot spot composed of an arginine-serine-arginine-serine-proline (RSRSP) stretch at aa 634–638 in the RS-rich region (Table 1; Figure 1B). This situation is unusual considering that most of the missense mutations were mapped to the RRM domains in our previous genetic screening for loss- or reduction-of-function mutants for splicing factors (Kuroyanagi et al., 2006, 2007, 2013). We will discuss later how these mutations would affect the function of RBM20.

**Table 1:** *RBM20* mutations identified in DCM patients and their symptoms other than ventricular dilatation.

**References**	**Domain**	**Mutation**	**Origin**	**Type**	**Effect on RBM20**	**Splicing regulation^a^**	**AF**	**VA**	**HF or SD**
Brauch et al., 2009	RS-rich	R634Q	Familial	Hetero	Unknown	Defective	0/10	1/10	0/10
		R636S	Familial	Hetero	Unknown	Defective	1/13	0/13	2/13
		R636H	Familial	Hetero	Unknown	Unknown	0/2	0/2	0/2
		S637G	Familial	Hetero	Unknown	Defective	0/4	1/4	1/4
		P638L	Familial	Hetero	Unknown	Defective	2/16	6/16	5/16
Li et al., 2010	RRM	V535I	Sporadic^b^	Hetero	Unknown	Unaffected	1/1	0/1	1/1
	RS-rich	R634Q	Sporadic	Hetero	Unknown	Defective	0/1	0/1	1/1
		R634W	Familial	Hetero	Exclusion from nucleus^c^	Defective	0/1	0/1	0/1
		R636C	Familial	Hetero	Unknown	Unknown	1/2	1/2	1/2
		R636H	Familial	Hetero	Unknown	Unknown	0/3	1/3	1/3
	Others	R716Q	Familial^d^	Hetero	Unknown	Defective	0/18	0/18	3/18
Millat et al., 2011	RS-rich	S637G	Familial	Hetero	Unknown	Unknown	Unknown	Unknown	Unknown
Refaat et al., 2012	L-rich	L83I	Sporadic	Hetero	Unknown	Unknown	0/1	0/1	Unknown
	RS-rich	P638L	Sporadic	Hetero	Unknown	Defective	1/1	0/1	Unknown
	E-rich	D888N	Sporadic	Hetero	Unaffected^c^	Unknown	1/1	0/1	Unknown
	Others	S455L	Sporadic	Hetero	Unknown	Unknown	0/1	0/1	Unknown
		R703S	Sporadic	Hetero	Unknown	Unknown	1/1	0/1	Unknown
		G1031X	Sporadic	Hetero	Unaffected^c^	Effective	0/1	0/1	Unknown
		P1081R	Sporadic	Hetero	Unaffected^c^	Unknown	0/1	0/1	Unknown
		E1206K	Sporadic	Hetero	Unaffected^c^	Unknown	0/1	0/1	Unknown
Guo et al., 2012	RS-rich	S635A	Sporadic	Hetero	Exclusion from nucleus^c^	Defective	Unknown	Unknown	Unknown
Wells et al., 2013	RS-rich	R636H	Familial	Hetero	Unknown	Unknown	Unknown	Unknown	Unknown
Chami et al., 2014	RS-rich	R636H	Familial	Hetero	Unknown	Unknown	Unknown	Unknown	Unknown
Zhao et al., 2015	Others	R1182H	Unknown	Hetero	Unknown	Unknown	Unknown	Unknown	Unknown
Beqqali et al., 2016	E-rich	E913K	Familial	Hetero	Protein destabilization	Defective	Unknown	Unknown	4/9
Murayama et al., 2018	RS-rich Others	R634W G1031X	Familial Sporadic^e^	Hetero Homo	Exclusion from nucleus^c^ Unaffected^c^	Defective Effective	0/2 1/2	0/2 1/2	1/2 1/2

*Note that residues within the RSRSP stretch in the RS-rich region are in red*.

a *Effect of RBM20 mutations on alternative splicing regulation of the TTN gene was assessed by using TTN splicing reporters (Guo et al., 2012; Murayama et al., 2018)*.

b *The patient also has an A698T mutation in LDB3*.

c *Effect of RBM20 mutations on RBM20 nuclear localization and phosphorylation of the RSRSP stretch was assessed by using mouse cDNAs with equivalent mutations (Murayama et al., 2018)*.

d *Incomplete penetrance*.

e *The patient is homozygous for the RBM20 mutation due to uniparental disomy, whereas his mother is heterozygous and asymptomatic*.

*AF, atrial fibrillation; HF, heart failure; SD, sudden death; VA, ventricular arrhythmias*.

## Alternative splicing of the *TTN* gene

**Figure 2:**
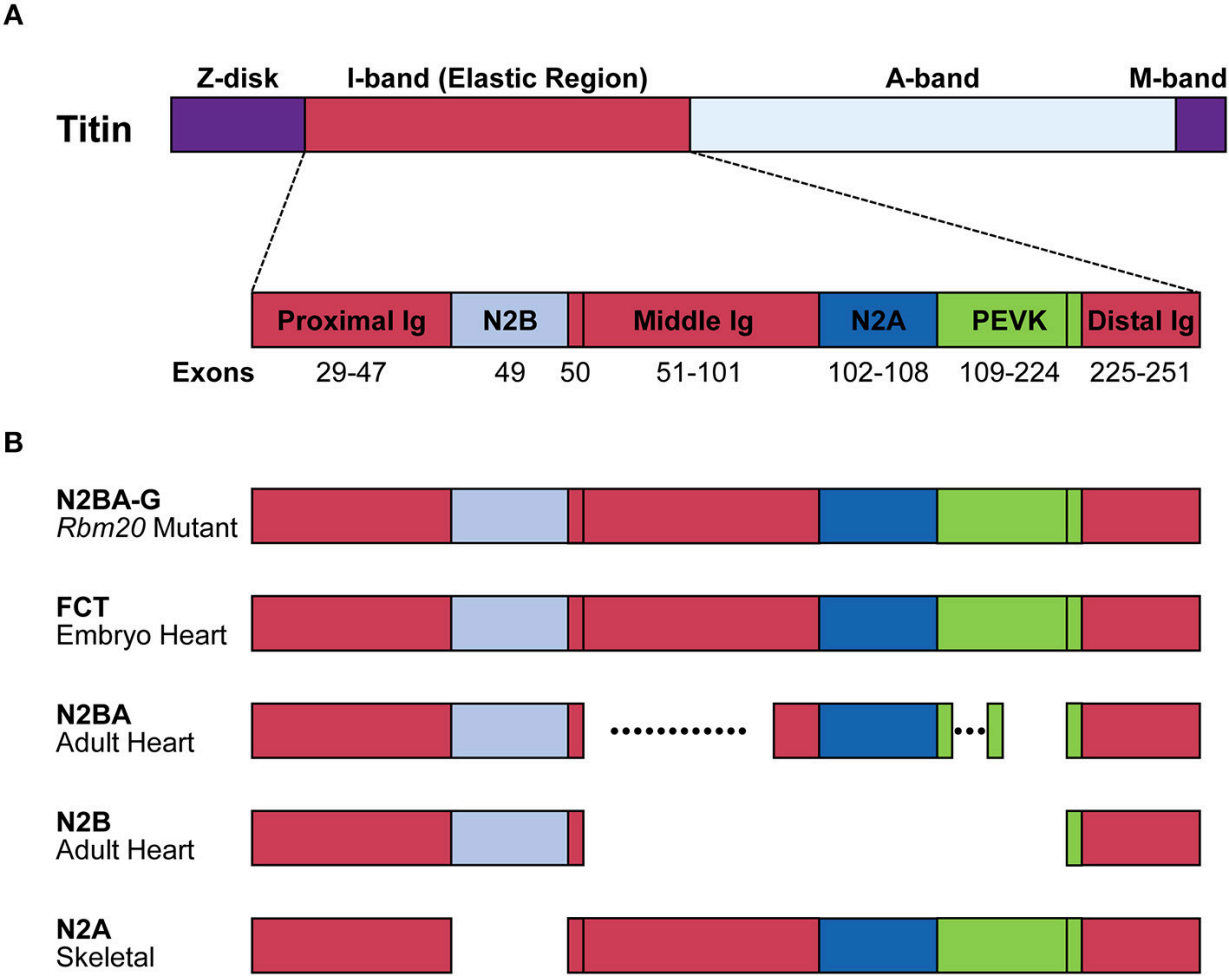
Structure of the titin protein isoforms. **(A)** Schematic domain structure of the titin protein. Names and positions of the domains are indicated. Corresponding exons are indicated below each domain. Distal Ig, distal Ig repeat domain; Middle Ig, middle Ig repeat domain; N2A, N2A-unique element; N2B, N2B-unique element; Proximal Ig, proximal Ig repeat domain. **(B)** Schematic structures of the titin isoforms. Names of the isoforms and the tissues that mainly express the isoforms are indicated on the left. Dotted lines indicate highly variable alternatively spliced regions. FCT, fetal cardiac titin.

Single titin protein spans half of sarcomere, with its N- and C-termini in the Z-disk and the M-band, respectively (Figure 2). It is composed of four structural and functional regions located in Z-disk, I-band, A-band, and M-band (Figure 2A; Wang et al., 1979). Titin is attached to the Z-disk and the thick filament via its Z-disk and A-band segments, respectively (Wang et al., 1979). The I-band region is not attached to any of the solid structures and therefore functions as a molecular spring that generates passive tension when sarcomeres are stretched during diastole (Horowits et al., 1986). In the elastic I-band region of titin, there are six domains from the N-terminus as follows: proximal immunoglobulin (Ig) repeat domain, N2B-unique element, middle Ig repeat domain, N2A-unique element, proline-glutamate-valine-lysine (PEVK) domain and distal Ig repeat domain (Figure 2A; Labeit et al., 1990; Bang et al., 2001; Lange et al., 2005).

The *TTN* gene can potentially produce an mRNA of more than 100 kb. Deduced from its sequence, the predicted full-length mRNA would produce a protein composed of ~39,000 amino acid residues whose molecular weight (MW) is 4.2 MDa (Bang et al., 2001; Guo and Sun, 2017). However, when SDS-PAGE was performed on rat myocardium samples, six titin isoforms of roughly 3.0 to 3.9 MDa were mainly identified (Zhu and Guo, 2017). While almost all exons encoding the Z-disk, A-band and M-band regions are constitutively included, many exons encoding the elastic I-band region are alternatively spliced in tissue-specific and developmentally regulated manners (Figure 2B). Exons encoding the proximal and distal Ig repeat domains are constitutively included in all isoforms. Exon 49, encoding the N2B-unique element, is a long exon (2,646 bp in human) and is specifically included in the heart but excluded in the skeletal muscle. Exon 50 is a constitutive exon and encodes an Ig domain. Exons 51–101 are alternatively spliced and encode the middle Ig repeat domain. Exons 102–108 and exons 109–224 encode the N2A-unique element and PEVK domain, respectively (Lewinter et al., 2007). Among these exons, alternative splicing of exons encoding the middle Ig repeat domain (exons 51–100) and the PEVK domain (exons 116–218) are exceptionally complicated (Guo et al., 2010). In the myocardium, two major titin isoforms are expressed; the shorter N2B isoform contains only the N2B-unique element among the variable domains; the longer N2BA isoforms including fetal types called fetal cardiac titin (FCT) contain the N2B- and N2A-unique elements and a variable length of the middle Ig repeat domain (Figure 1). In the skeletal muscle, the N2A isoform containing all of the variable domains except for the N2B-unique element is expressed (Lewinter et al., 2007; Guo and Sun, 2017; Figure 2B).

RBM20 is the major regulator of the heart-specific *TTN* pre-mRNA splicing. In the spontaneous *Rbm20* mutant rat strain lacking a 95-kb region spanning from exons 2–14, titin N2B is no longer expressed while N2BA is predominantly expressed in heterozygotes, and an extraordinarily large isoform N2BA-G is exclusively expressed in homozygotes (Guo et al., 2012); Figure 2B. The N2BA isoform is also predominantly expressed in the heart of a DCM patient carrying a heterozygous missense mutation *S635A* in the *RBM20* gene (Guo et al., 2012). In a human heart-failure cohort, low expression of endogenous RBM20 was correlated with the splicing pattern of the *TTN* gene (Maatz et al., 2014). These reports indicated that splicing control of many of the *TTN* exons is extremely sensitive to the amount of functional RBM20 protein.

## RBM20 regulates heart-specific alternative splicing

High-throughput RNA sequencing (RNA-seq) of cardiac transcriptomes from the *Rbm20*-null rats and human DCM patients with and without mutations in *RBM20* revealed 31 genes whose alternative splicing is RBM20-dependent in both rats and humans (Guo et al., 2012). Crosslinking and immunoprecipitation coupled with RNA-seq (CLIP-seq) experiments of endogenous RBM20 in rat cardiomyocytes identified 80 direct target exons in 18 genes (Maatz et al., 2014). RBM20 predominantly represses cassette exons including those in the *Ttn* and *Ryr2* genes by binding to upstream and/or downstream intron(s) of the target exons (Li et al., 2013; Maatz et al., 2014). Mutually exclusive exons are also enriched among the RBM20 target exons (Guo et al., 2012; Maatz et al., 2014). For instance, RBM20 represses exons 15 and 16 and promotes inclusion of exon 14 in the *Camk2d* gene encoding Ca^2+^/calmodulin-dependent protein kinase II-δ (CaMKII-δ); RBM20 represses exons 5–7 and promotes inclusion of exon 4 in the *Ldb3* gene encoding Lim domain binding protein 3 (LDB3).

A UCUU core element has been identified as a precise RNA recognition element (RRE) for RBM20 by photoactivatable ribonucleoside-enhanced crosslinking and immunoprecipitation (PAR-CLIP) experiments with epitope-tagged human RBM20 in human embryonic kidney 293 (HEK293) cells and by the CLIP-seq experiments with rat cardiomyocytes (Maatz et al., 2014). In the *Ttn* pre-mRNA, RBM20-binding sites were identified in many of the introns between exon 50 and exon 219 but were almost excluded from the constitutively spliced regions (Maatz et al., 2014). The introns in the alternatively spliced regions were retained in the wild-type rat heart (Li et al., 2013), suggesting that RBM20 represses exons 51–218 by inhibiting excision of most if not all of the introns in this region.

## The RSRSP stretch in the RS-rich region is crucial for nuclear localization of RBM20

**Figure 3:**
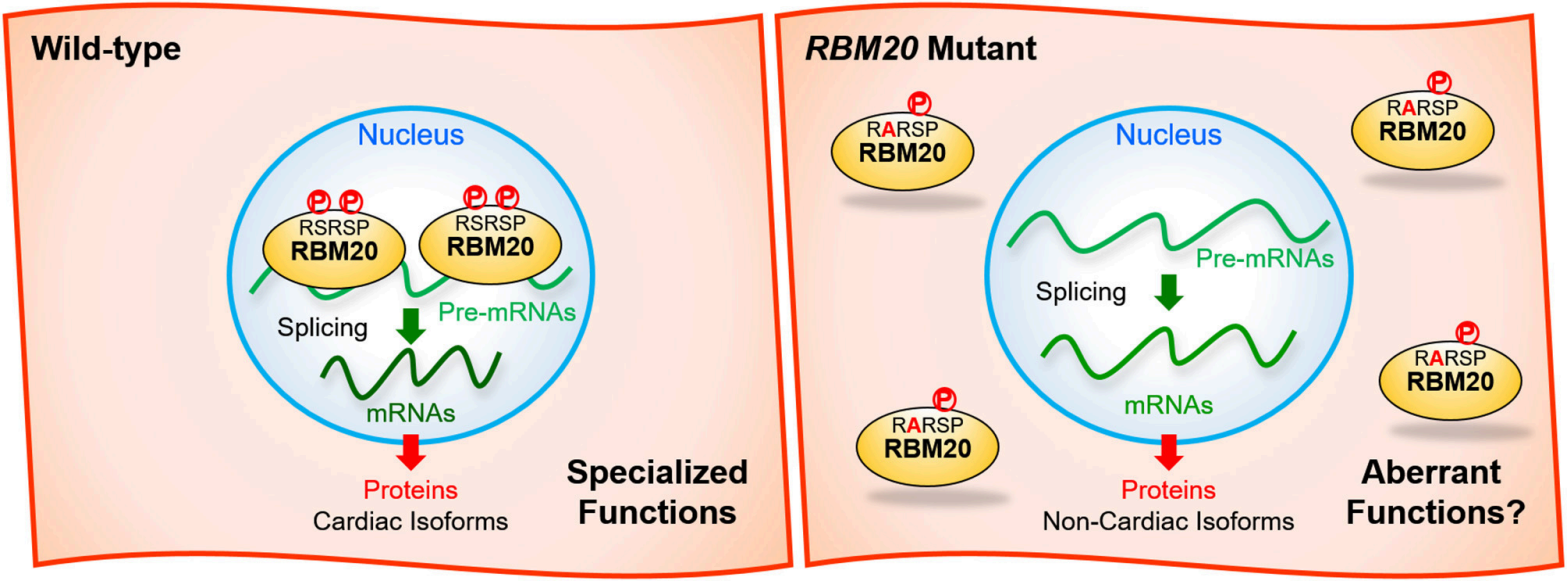
Missense mutations in the RSRSP stretch disrupt the normal functions of RBM20. (Left) In the wild-type, two serine residues in the RSRSP stretch are phosphorylated and the RBM20 protein is localized in the nucleus, where RBM20 regulates alternative pre-mRNA splicing of its target genes so that cardiac isoforms of mRNAs are produced. The mRNAs are translated into cardiac protein isoforms with specialized functions. (Right) In the *RBM20* missense mutant with a substitution in the RSRSP stretch, the mutant RBM20 proteins are no longer imported into the nucleus. Pre-mRNAs of the RBM20-target genes are processed into non-cardiac isoforms of mRNAs, which are then translated into non-cardiac protein isoforms, which may lack the specialized functions and/or exert aberrant functions. The mutant RBM20 proteins retained in the cytoplasm may also exert aberrant functions. P, phosphorylation.

Recently, it has been reported that both of the two serine residues in the RSRSP stretch are constitutively phosphorylated in cells and that single amino acid substitutions in the stretch disrupted nuclear localization of full-length RBM20 protein (Murayama et al., 2018; Figure 3). Moreover, *Rbm20*^*S*637*A*^ knock-in mouse mimicking the S635A mutation showed a remarkable effect on the titin isoform expression like in the *Rbm20*-null rat strain (Murayama et al., 2018). These findings indicated that the RSRSP stretch is a crucial part of the RBM20 nuclear localization signal (NLS) and that the DCM-linked mutation in the RSRSP stretch fully disrupted the alternative splicing control by RBM20.

Many of splicing factors such as SR-protein family members are known to have RS-rich regions or RS domains consisting of multiple serine-arginine (SR) and arginine-serine (RS) dipeptides (Zahler et al., 1992). The RS domains of the SR proteins are extensively phosphorylated on the serine residues and this phosphorylation plays an important role in regulating subcellular localization, protein-protein interaction and splicing regulation activities of the SR proteins (Xiao and Manley, 1997; Yeakley et al., 1999). It is therefore reasonable to suggest that the RBM20-mediated heart-specific alternative splicing is dynamically regulated during development and under pathological conditions via dynamic phosphorylation/dephosphorylation of the RSRSP stretch.

Many RBM20-interacting proteins have been identified in HEK293 cells by quantitative stable isotope labeling by amino acids in cell culture (SILAC)-based proteomics experiments and some of these interactions were affected by the S635A mutation (Maatz et al., 2014). Totally distinct subcellular localization of the wild-type and mutant RBM20 proteins (Murayama et al., 2018) might be the major cause of this distinct interactomes. Even with the information about the RBM20 interactome, it is still unclear how the interaction with these proteins leads to repression or switching of its target exons.

## The RRM domain of RBM20 is crucial for splicing regulation *in vivo*

Molecular mechanisms of RBM20-mediated alternative splicing have been analyzed mostly by utilizing *TTN* reporter minigenes expressed in non-cardiac cells. However, the results from such studies utilizing distinct reporter minigenes are controversial as to which of the conserved domains are crucial for the regulation. Mutations in the RSRSP stretch but not in the RRM domain affected repression of the 5′ PEVK exons (Guo et al., 2012). The RRM domain and the E-rich region were crucial for repressing exon 242 (Liss et al., 2018). The RSRSP stretch and the E-rich region but not putative RNA-binding domains RRM, ZnF1, or ZnF2 were crucial for repression of a chimeric exon 51/218 (Murayama et al., 2018). Full-length RBM20 (Maatz et al., 2014) or the RRM domain alone (Dauksaite and Gotthardt, 2018) does not necessarily bind to any RNA molecules containing UCUU element(s) in electrophoretic mobility shift assays (EMSAs). Therefore, other RNA element(s) and/or other RNA-binding protein(s) might be involved in the recognition of the authentic target pre-mRNAs by RBM20.

Functions of the RRM domain *in vivo* has been assessed by deleting exons 6 and 7 of the *Rbm20* gene in the mouse. N2BA and N2BA-G isoforms of titin proteins predominated in the left ventricles (LVs) of the *Rbm20*^Δ*RRM*^ heterozygotes and homozygotes, respectively, and alternative splicing of the *Camk2d* and *Ldb3* genes was evidently affected as in the *Rbm20*-deficient rats (Methawasin et al., 2014), indicating that the RRM domain is crucial for the splicing regulation of these genes *in vivo*. However, the heterozygous or homozygous *Rbm20*^Δ*RRM*^ mice did not show any significant differences in cardiac chamber geometry and dimensions compared to wild-type controls (Methawasin et al., 2014), suggesting that switching of the titin isoforms to N2BA-G together with the splicing change in *Camk2d* and *Ldb3 per se* does not cause DCM-like phenotypes such as LV chamber dilatation and systolic dysfunction. This is consistent with that there is not a reported familial case where a missense mutation is mapped to the RRM domain (Table 1). Interestingly, the phenotypes of the *Rbm20*^Δ*RRM*^ mice are distinct from those of the *Rbm20*-deficient rats (Guo et al., 2012) and *Rbm20* knockout (KO) mice in which exons 4 and 5 are deleted for a frame-shift (van den Hoogenhof et al., 2018): the heterozygous and homozygous *Rbm20* null rats and mice showed LV dilatation in addition to drastic splicing changes in the *Ttn, Camk2d* and *Ldb3* genes. These observations suggest that RBM20(ΔRRM) protein retains some regulatory functions. Elucidation of transcripts differentially affected between the *Rbm20*^Δ*RRM*^ and *Rbm20* KO mice would lead to identification of genes crucial for the progression of the DCM-like phenotypes in the rodent models.

The only *RBM20* missense mutation outside of the RSRSP stretch in familial DCM cases with complete penetrance was mapped to a highly conserved glutamate (E) residue in the E-rich region (Beqqali et al., 2016; van den Hoogenhof et al., 2018; Table 1). The E913K mutation has been shown to decrease the amount of total RBM20 protein and to affect the *TTN* splicing in the heart of a patient heterozygous for the mutation (Beqqali et al., 2016), suggesting that the E-rich region is crucial for the stability of RBM20. Other missense mutations outside of the RSRSP stretch were identified only in sporadic cases (Table 1) and no experimental evidence of altered splicing has been demonstrated. Therefore, it is unclear whether these mutations affected RBM20 functions and hence caused the DCM phenotypes.

The only *RBM20* non-sense mutation in the DCM patients reported so far is G1031X in sporadic cases (Table 1). This mutation is in exon 11 and likely causes non-sense-mediated mRNA decay (NMD) of mature mRNAs (Schweingruber et al., 2013), leading to haploinsufficiency of *RBM20*. Notably, one of the patients is homozygous for the G1031X mutation due to uniparental disomy, whereas his mother is asymptomatic even with the heterozygous G1031X mutation (Murayama et al., 2018). It is therefore under debate whether heterozygous non-sense mutations in *RBM20* leading to haploinsufficiency would cause the DCM phenotypes.

## Analysis of the RBM20 functions with pluripotent stem cells

Expression profiling throughout *in vitro* cardiogenesis in embryoid bodies (EBs) derived from mouse embryonic stem cells (mESCs) revealed that *Rbm20* became expressed as early as *Nkx2-5*, a marker for cardiac progenitors, consistent with *Rbm20* induction during *in vivo* cardiogenesis between E7.5 and E8.5 (Beraldi et al., 2014). Even though *Rbm20* is maximally expressed at day 9 of *in vitro* differentiation, the transition of the titin isoforms was apparent at day 24, which was suppressed by *Rbm20* knockdown (Beraldi et al., 2014), suggesting that RBM20-mediated splicing regulation is reproduced in the *in vitro* cardiogenesis.

*In vitro* differentiation of human induced-pluripotent stem cell (hiPSC)-derived cardiomyocytes (hiPSC-CMs) from familial DCM patients carrying different *RBM20* mutations have been utilized for gene expression profiling during *in vitro* cardiogenesis. Cytological analysis of the hiPSC-CMs revealed that the *RBM20* mutations disorganized sarcomere structures (Wyles et al., 2016b; Streckfuss-Bömeke et al., 2017). The *RBM20* hiPSC-CMs were defective in Ca^2+^ handling machinery with prolonged Ca^2+^ levels in the cytoplasm and higher Ca^2+^ spike amplitude (Wyles et al., 2016b; Streckfuss-Bömeke et al., 2017), consistent with an increased risk of malignant ventricular arrhythmias in DCM patients with *RBM20* mutations than those with *TTN* mutations (van den Hoogenhof et al., 2018). The *RBM20* hiPSC-CMs have also been utilized for demonstrating their increased susceptibility to β-adrenergic stress and therapeutic rescue by a β-blocker carvedilol and a Ca^2+^ channel blocker verapamil (Wyles et al., 2016a).

## Regulation of titin compliance by RBM20

Titin-based passive tension in the cardiomyocytes occupies a large proportion of the passive stiffness of the whole myocardium (Rivas-Pardo et al., 2016). It has a negative correlation with molecular weight or amino acid sequence length of titin's spring region. For instance, N2B has a shorter elastic region compared to N2BA, thus giving higher passive stiffness to the cardiomyocytes. It is therefore believed that the ratio of the titin isoforms as well as the total amount of titin protein influence the myocardial passive stiffness (Lahmers et al., 2004). The ratio of the N2B isoform to the N2BA isoform varies from species to species (Neagoe et al., 2003) and from ventricles to atria (Fukuda et al., 2003). It is also dynamically regulated during development (Lahmers et al., 2004; Opitz et al., 2004; Warren et al., 2004; Opitz and Linke, 2005; Figure 2B) and under pathological conditions. Amount of the N2BA isoform was increased in heart failure with DCM, heart failure with reduced ejection fraction (HFrEF) and chronic ischemic cardiomyopathy (Makarenko et al., 2004; Nagueh et al., 2004; Borbély et al., 2008), and a reduction of the N2BA isoform was observed in diastolic dysfunction resulting from hypertensive heart disease (Warren et al., 2003).

In the *Rbm20*^Δ*RRM*^ heterozygote mice, the compliance of titin proteins is increased and diastolic stiffness of a LV chamber is reduced without significant effect on the chamber geometry or dimensions; beneficial effects on diastolic function dominated under conditions of exercise over an unfavorable effect on end-systolic elastance (Methawasin et al., 2014). Splicing changes in the *Ldb3* and *Camk2d* genes in the *Rbm20*^Δ*RRM*^ heterozygotes caused minimal effects on the LDB3 isoforms and no apparent effects on phosphorylation of known CaMKII-δ targets (Methawasin et al., 2014). The *Rbm20*^Δ*RRM*^ mice have therefore been utilized to prove a concept that increasing the titin compliance is beneficial to disease models where the mice suffer from lowered diastolic stiffness of the hearts (see below).

## RBM20 as a potential therapeutic target

Heart failure with preserved ejection fraction (HFpEF) is a complex syndrome that includes diastolic dysfunction, exercise intolerance and concentric hypertrophic remodeling. Deletion of *Ttn* exons 251–269, corresponding to the I-band–A-band junction (IAjxn) of titin, increases strain on the spring region and causes an HFpEF-like syndrome in mice (Granzier et al., 2014). Upon constitutive or inducible, heart-specific heterozygous deletion of the RBM20 RRM domain in the *Ttn*^Δ*IAjxn*^ mice, compliant titin isoforms were expressed, diastolic function was normalized, exercise performance was improved and pathological hypertrophy was attenuated (Bull et al., 2016). HFpEF model mice can also be prepared by performing transverse aortic constriction (TAC) surgery with deoxycorticosterone acetate (DOCA) pellet implantation; inducible, heart-specific heterozygous deletion of the RRM domain in this model mice has also been shown to ameliorate diastolic dysfunction and to recover exercise intolerance (Methawasin et al., 2016).

Deletion of *Ttn* exon 49, corresponding to the N2B-unique element, results in small hearts with reduced sarcomere length and increased passive tension leading to diastolic dysfunction in mice (Radke et al., 2007). Heterozygous deletion of the RBM20 RRM domain from the *Ttn* N2B KO mice restored the cardiac dimension and improved the diastolic function (Hinze et al., 2016).

Deletion of *Ttn* constitutive exons 30–38, corresponding to nine proximal Ig domains in the spring region, increased diastolic stiffness leading to diastolic dysfunction (Chung et al., 2013) and caused mild kyphosis, a phenotype associated with skeletal muscle myopathy (Buck et al., 2014) in mice. RBM20 was upregulated at the protein level in the *Ttn* IG KO soleus muscle leading to further shortening of titin and heterozygous deletion of the RBM20 RRM domain from the *Ttn* IG KO mice restored the length of titin in the soleus (Buck et al., 2014).

These examples demonstrated that diastolic dysfunctions could be rescued by increasing titin compliance through manipulation of *Ttn* pre-mRNA splicing in the animal models, and raised the *Ttn* splicing regulator RBM20 as a potential therapeutic target. Recently, high-throughput screening of small compounds were performed with RBM20-sensitive *TTN* splicing reporters and cardenolides have been shown to inhibit RBM20-mediated repression of *TTN* exons at least in part by reducing RBM20 protein level in cultured cells (Liss et al., 2018).

Extracellular and intracellular signals affecting the titin isoform ratios via RBM20 have also been investigated. Thyroid hormone T3 can promote developmental titin isoform transitions in primary rat cardiomyocytes via the phosphatidylinositol-3-kinase (PI3K)/AKT pathway (Krüger et al., 2008) and the effect of T3 on titin is dependent on RBM20 (Zhu et al., 2015). Western blot analysis of the cardiomyocytes with anti-RBM20 antibody detected two bands with apparent molecular weight of 135 kDa and 145 kDa and the amounts of the two isoforms were differentially affected by T3 and/or a PI3K inhibitor, implying that the activity of RBM20 is regulated by post-translational modification(s) (Zhu et al., 2015). Insulin can also promote the developmental transition of the titin isoforms in primary rat cardiomyocytes by increasing the amount of RBM20 proteins through the PI3K-Akt-mTOR kinase axis (Zhu et al., 2017).

## Regulation of circular rna production from the *TTN* gene by RBM20

Circular RNAs (circRNAs) can be generated by “back-splicing” of pre-mRNAs (Li et al., 2018) and some of the circRNAs have physiological functions as miRNA sponges *in vivo* (Hansen et al., 2013; Memczak et al., 2013). The *TTN* gene has been shown to produce a variety of circRNAs mostly from the alternatively spliced exons (Li et al., 2013; Khan et al., 2016). Recent RNA-seq experiments identified thousands of circRNAs in the human heart (Khan et al., 2016) and >1,000 circRNAs in the mouse heart (Aufiero et al., 2018). However, only a small subset of circRNAs expressed in the heart are evolutionarily conserved (Aufiero et al., 2018), implying that most of the cardiac circRNAs are non-functional. Forty-three of the human cardiac circRNAs including those from the *TTN* and *CAMK2D* genes were differentially expressed in heart samples from DCM patients compared with those from controls (Khan et al., 2016). Thirty-eight of the mouse cardiac circRNAs were differentially expressed in the *Rbm20* KO mice, 11 of which were generated from the *Ttn* gene in an RBM20-dependent manner (Aufiero et al., 2018). One may therefore say that RBM20 switches from N2BA-G production to circRNA production from the *TTN* gene, although physiological and pathological functions of the circRNAs from the *TTN* and other genes remain to be elucidated.

## Open questions and future research directions

Although it has been genetically shown that the *RBM20* mutations cause DCM, subsequent cardiac transcriptome analyses and animal models have not yet specified RBM20-regulated genes whose aberrant splicing are critically linked to each of the DCM symptoms such as systolic dysfunction, left ventricle dilatation and a risk of ventricular arrhythmia. RBM20 mutant protein with a missense mutation in the RSRSP stretch may exert aberrant functions in the cytoplasm (Figure 3). Identification of such critical splice variants or aberrant effects may lead to development of new therapeutics for DCM symptoms not restricted to those caused by the *RBM20* mutations. To validate the candidate events, it is necessary to genetically restore cardiac isoforms and/or reduce aberrant isoforms in an appropriate animal model that show DCM-like phenotypes as in the *RBM20*-linked DCM patients. Ventricular arrhythmia has not been reported for the Δ*RRM* or *KO* mouse models, and therefore another animal model that phenocopies the *RBM20*-linked DCM is awaited. A recent large-scale genome-wide association study (GWAS) of >1 million people including 60,620 atrial fibrillation cases have identified *RBM20* as one of genes near risk variants (Nielsen et al., 2018), suggesting its implication in atrial cardiomyopathy.

*TTN* exons 51–124 and alternative splicing events in *CAMK2D* and *LDB3* are hyper-sensitive to reduction in the amount of functional RBM20 protein, whereas *TTN* exons 125–218 are much less affected by the heterozygous mutations (Guo et al., 2012; Beqqali et al., 2016; Murayama et al., 2018). Biochemical and biophysical analysis of the splicing control of *TTN* by RBM20 should address the following points: (1) how tens of consecutive cassette exons can be synchronously repressed depending on the amount of a single factor RBM20, and (2) how other tens of consecutive cassette exons can be almost completely repressed by less amount of RBM20. Application of recent technical progress in direct sequencing of full-length cDNA/mRNA to the extremely long *TTN* transcripts would elucidate precise splicing patterns of the RBM20-dependent isoforms, which will help understanding the regulation mechanisms.

RBM20 is expressed at early cardiogenesis, yet a compliant titin FCT isoform is expressed in the embryonic heart. RBM20 is also expressed in the skeletal muscle, yet the splicing patterns of the *TTN* mRNAs are totally different between these tissues. So far, it is unknown how the activity of RBM20 is regulated during development and in different tissues. Elucidating such mechanisms will lead to further understanding of heart-specific alternative splicing regulation as well as to future possible therapeutics that manipulate the activity of RBM20.

## Author contributions

TW, AK, and HK drafted the manuscript, and revised critically the manuscript for important intellectual content.

### Conflict of interest statement

The authors declare that the research was conducted in the absence of any commercial or financial relationships that could be construed as a potential conflict of interest.

## References

[B1] Aufiero S. van den Hoogenhof M. M. G. Reckman Y. J. Beqqali A. van der Made I. Kluin J.. (2018). Cardiac circRNAs arise mainly from constitutive exons rather than alternatively spliced exons. RNA 24, 815–827. 10.1261/rna.064394.117 29567830 PMC5959250

[B2] Bang M. L. Centner T. Fornoff F. Geach A. J. Gotthardt M. McNabb M.. (2001). The complete gene sequence of titin, expression of an unusual approximately 700-kDa titin isoform, and its interaction with obscurin identify a novel Z-line to I-band linking system. Circ. Res. 89, 1065–1072. 10.1161/hh2301.100981 11717165

[B3] Beqqali A. Bollen I. A. E. Rasmussen T. B. van den Hoogenhof M. M. van Deutekom H. W. M. Schafer S.. (2016). A mutation in the glutamate-rich region of RNA-binding motif protein 20 causes dilated cardiomyopathy through missplicing of titin and impaired Frank–Starling mechanism. Cardiovasc. Res. 112, 452–463. 10.1093/cvr/cvw192 27496873

[B4] Beraldi R. Li X. Martinez Fernandez A. Reyes S. Secreto F. Terzic A.. (2014). Rbm20-deficient cardiogenesis reveals early disruption of RNA processing and sarcomere remodeling establishing a developmental etiology for dilated cardiomyopathy. Hum. Mol. Genet. 23, 3779–3791. 10.1093/hmg/ddu091 24584570 PMC4065152

[B5] Borbély A. van Heerebeek L. Paulus W. J. (2008). Transcriptional and Posttranslational Modifications of Titin. Circ. Res. 104, 12–14. 10.1161/CIRCRESAHA.108.191130 19118283

[B6] Brauch K. M. Karst M. L. Herron K. J. de Andrade M. Pellikka P. A. Rodeheffer R. J.. (2009). Mutations in ribonucleic acid binding protein gene cause familial dilated cardiomyopathy. J. Am. Coll. Cardiol. 54, 930–941. 10.1016/j.jacc.2009.05.038 19712804 PMC2782634

[B7] Buck D. Smith J. E. Chung C. S. Ono Y. Sorimachi H. Labeit S.. (2014). Removal of immunoglobulin-like domains from titin's spring segment alters titin splicing in mouse skeletal muscle and causes myopathy. J. Gen. Physiol. 143, 215–230. 10.1085/jgp.201311129 24470489 PMC4001778

[B8] Bull M. Methawasin M. Strom J. Nair P. Hutchinson K. Granzier H. (2016). Alternative splicing of titin restores diastolic function in an HFpEF-like genetic murine model (*Ttn* ^Δ*IAjxn*^)novelty and significance. Circ. Res. 119, 764–772. 10.1161/CIRCRESAHA.116.308904 27470639 PMC5059842

[B9] Chami N. Tadros R. Lemarbre F. Lo K. S. Beaudoin M. Robb L.. (2014). Nonsense mutations in BAG3 are associated with early-onset dilated cardiomyopathy in French Canadians. Can. J. Cardiol. 30, 1655–1661. 10.1016/j.cjca.2014.09.030 25448463

[B10] Chung C. S. Hutchinson K. R. Methawasin M. Saripalli C. Smith J. E. Hidalgo C. G.. (2013). Shortening of the elastic tandem immunoglobulin segment of titin leads to diastolic dysfunction. Circulation 128, 19–28. 10.1161/CIRCULATIONAHA.112.001268 23709671 PMC3822017

[B11] Coelho M. B. Attig J. Ule J. Smith C. W. J. (2016). Matrin3: connecting gene expression with the nuclear matrix. Wiley Interdiscip. Rev. RNA 7, 303–315. 10.1002/wrna.1336 26813864

[B12] Dadson K. Hauck L. Billia F. (2017). Molecular mechanisms in cardiomyopathy. Clin. Sci. 131, 1375–1392. 10.1042/CS20160170 28645928

[B13] Dauksaite V. Gotthardt M. (2018). Molecular basis of titin exon exclusion by RBM20 and the novel titin splice regulator PTB4. Nucleic Acids Res. 46, 5227–5238. 10.1093/nar/gky165 29518215 PMC6007684

[B14] Elliott P. M. (2014). 2014 ESC Guidelines on diagnosis and management of hypertrophic cardiomyopathy. Eur. Heart J. 35, 2733–2779 10.1093/eurheartj/ehu284 25173338

[B15] Fatkin D. Huttner I. G. (2017). Titin-truncating mutations in dilated cardiomyopathy. Curr. Opin. Cardiol. 32, 232–238. 10.1097/HCO.0000000000000382 28151760

[B16] Filippello A. Lorenzi P. Bergamo E. Romanelli M. G. (2013). Identification of nuclear retention domains in the RBM20 protein. FEBS Lett. 587, 2989–2995. 10.1016/j.febslet.2013.07.018 23886709

[B17] Fukuda N. Wu Y. Farman G. Irving T. C. Granzier H. (2003). Titin isoform variance and length dependence of activation in skinned bovine cardiac muscle. J. Physiol. 553, 147–154. 10.1113/jphysiol.2003.049759 12963792 PMC2343481

[B18] Gigli M. Begay R. L. Morea G. Graw S. L. Sinagra G. Taylor M. R. G.. (2016). A review of the giant protein titin in clinical molecular diagnostics of cardiomyopathies. Front. Cardiovasc. Med. 3:21. 10.3389/fcvm.2016.00021 27493940 PMC4954824

[B19] Granzier H. L. Hutchinson K. R. Tonino P. Methawasin M. Li F. W. Slater R. E.. (2014). Deleting titin's I-band/A-band junction reveals critical roles for titin in biomechanical sensing and cardiac function. Proc. Natl. Acad. Sci. U.S.A. 111, 14589–14594. 10.1073/pnas.1411493111 25246556 PMC4210014

[B20] Guo W. Bharmal S. J. Esbona K. Greaser M. L. (2010). Titin diversity–alternative splicing gone wild. J. Biomed. Biotechnol. 2010:753675. 10.1155/2010/753675 20339475 PMC2843904

[B21] Guo W. Schafer S. Greaser M. L. Radke M. H. Liss M. Govindarajan T.. (2012). RBM20, a gene for hereditary cardiomyopathy, regulates titin splicing. Nat. Med. 18, 766–773. 10.1038/nm.2693 22466703 PMC3569865

[B22] Guo W. Sun M. (2017). RBM20, a potential target for treatment of cardiomyopathy via titin isoform switching. Biophys. Rev. 10, 15–25. 10.1007/s12551-017-0267-5 28577155 PMC5803173

[B23] Hansen T. B. Jensen T. I. Clausen B. H. Bramsen J. B. Finsen B. Damgaard C. K.. (2013). Natural RNA circles function as efficient microRNA sponges. Nature 495, 384–388. 10.1038/nature11993 23446346

[B24] Herman D. S. Lam L. Taylor M. R. G. Wang L. Teekakirikul P. Christodoulou D.. (2012). Truncations of titin causing dilated cardiomyopathy. N. Engl. J. Med. 366, 619–628. 10.1056/NEJMoa1110186 22335739 PMC3660031

[B25] Hershberger R. E. Hedges D. J. Morales A. (2013). Dilated cardiomyopathy: the complexity of a diverse genetic architecture. Nat. Rev. Cardiol. 10, 531–547. 10.1038/nrcardio.2013.105 23900355

[B26] Hidalgo C. Granzier H. (2013). Tuning the molecular giant titin through phosphorylation: role in health and disease. Trends Cardiovasc. Med. 23, 165–171. 10.1016/j.tcm.2012.10.005 23295080 PMC3622841

[B27] Hinze F. Dieterich C. Radke M. H. Granzier H. Gotthardt M. (2016). Reducing RBM20 activity improves diastolic dysfunction and cardiac atrophy. J. Mol. Med. 94, 1349–1358. 10.1007/s00109-016-1483-3 27889803 PMC5143357

[B28] Horowits R. Kempner E. S. Bisher M. E. Podolsky R. J. (1986). A physiological role for titin and nebulin in skeletal muscle. Nature 323, 160–164. 10.1038/323160a0 3755803

[B29] Kayvanpour E. Sedaghat-Hamedani F. Amr A. Lai A. Haas J. Holzer D. B.. (2017). Genotype-phenotype associations in dilated cardiomyopathy: meta-analysis on more than 8000 individuals. Clin. Res. Cardiol. 106, 127–139. 10.1007/s00392-016-1033-6 27576561

[B30] Khan M. A. Reckman Y. J. Aufiero S. van den Hoogenhof M. M. van der Made I. Beqqali A.. (2016). RBM20 regulates circular RNA production from the titin gene. Circ. Res. 119, 996–1003. 10.1161/CIRCRESAHA.116.309568 27531932

[B31] Kimura A. (2016). Molecular genetics and pathogenesis of cardiomyopathy. J. Hum. Genet. 61, 41–50. 10.1038/jhg.2015.83 26178429

[B32] Kirk R. Naftel D. Hoffman T. M. Almond C. Boyle G. Caldwell R. L.. (2009). Outcome of pediatric patients with dilated cardiomyopathy listed for transplant: a multi-institutional study. J. Heart Lung Transplant. 28, 1322–1328. 10.1016/j.healun.2009.05.027 19782601 PMC4296522

[B33] Krüger M. Sachse C. Zimmermann W. H. Eschenhagen T. Klede S. Linke W. A. (2008). Thyroid hormone regulates developmental titin isoform transitions via the phosphatidylinositol-3-kinase/ AKT pathway. Circ. Res. 102, 439–447. 10.1161/CIRCRESAHA.107.162719 18096819

[B34] Kuroyanagi H. Kobayashi T. Mitani S. Hagiwara M. (2006). Transgenic alternative-splicing reporters reveal tissue-specific expression profiles and regulation mechanisms *in vivo*. Nat. Methods 3, 909–915. 10.1038/nmeth944 17060915

[B35] Kuroyanagi H. Ohno G. Mitani S. Hagiwara M. (2007). The Fox-1 family and SUP-12 coordinately regulate tissue-specific alternative splicing *in vivo*. Mol. Cell. Biol. 27, 8612–8621. 10.1128/MCB.01508-07 17923701 PMC2169414

[B36] Kuroyanagi H. Watanabe Y. Hagiwara M. (2013). CELF family RNA–binding protein UNC-75 regulates two sets of mutually exclusive exons of the unc-32 gene in neuron-specific manners in *Caenorhabditis elegans*. PLoS Genet. 9:e1003337. 10.1371/journal.pgen.1003337 23468662 PMC3585155

[B37] Labeit S. Barlow D. P. Gautel M. Gibson T. Holt J. Hsieh C.-L.. (1990). A regular pattern of two types of 100-residue motif in the sequence of titin. Nature 345, 273–276. 10.1038/345273a0 2129545

[B38] Lahmers S. Wu Y. Call D. R. Labeit S. Granzier H. (2004). Developmental control of titin isoform expression and passive stiffness in fetal and neonatal myocardium. Circ. Res. 94, 505–513. 10.1161/01.RES.0000115522.52554.86 14707027

[B39] Lange S. Xiang F. Yakovenko A. Vihola A. Hackman P. Rostkova E.. (2005). The kinase domain of titin controls muscle gene expression and protein turnover. Science 308, 1599–1603. 10.1126/science.1110463 15802564

[B40] Lewinter M. M. Wu Y. Labeit S. Granzier H. (2007). Cardiac titin: Structure, functions and role in disease. Clin. Chim. Acta 375, 1–9. 10.1016/j.cca.2006.06.035 16904093

[B41] Li D. Morales A. Gonzalez-Quintana J. Norton N. Siegfried J. D. Hofmeyer M.. (2010). Identification of novel mutations in RBM20 in patients with dilated cardiomyopathy. Clin. Transl. Sci. 3, 90–97. 10.1111/j.1752-8062.2010.00198.x 20590677 PMC2898174

[B42] Li S. Guo W. Dewey C. N. Greaser M. L. (2013). Rbm20 regulates titin alternative splicing as a splicing repressor. Nucleic Acids Res. 41, 2659–2672. 10.1093/nar/gks1362 23307558 PMC3575840

[B43] Li X. Yang L. Chen L.-L. (2018). The biogenesis, functions, and challenges of circular RNAs. Mol. Cell 71, 428–442. 10.1016/j.molcel.2018.06.034 30057200

[B44] Liss M. Radke M. H. Eckhard J. Neuenschwander M. Dauksaite V. von Kries J.-P.. (2018). Drug discovery with an RBM20 dependent titin splice reporter identifies cardenolides as lead structures to improve cardiac filling. PLoS ONE 13:e0198492. 10.1371/journal.pone.0198492 29889873 PMC5995442

[B45] Maatz H. Jens M. Liss M. Schafer S. Heinig M. Kirchner M.. (2014). RNA-binding protein RBM20 represses splicing to orchestrate cardiac pre-mRNA processing. J. Clin. Invest. 124, 3419–3430. 10.1172/JCI74523 24960161 PMC4109538

[B46] Makarenko I. Opitz C. A. Leake M. C. Neagoe C. Kulke M. Gwathmey J. K.. (2004). Passive stiffness changes caused by upregulation of compliant titin isoforms in human dilated cardiomyopathy hearts. Circ. Res. 95, 708–716. 10.1161/01.RES.0000143901.37063.2f 15345656

[B47] McCartan C. Mason R. Jayasinghe S. R. Griffiths L. R. (2012). Cardiomyopathy classification: ongoing debate in the genomics era. Biochem. Res. Int. 2012, 1–10. 10.1155/2012/796926 22924131 PMC3423823

[B48] McKenna W. J. Maron B. J. Thiene G. (2017). Classification, epidemiology, and global burden of cardiomyopathies. Circ. Res. 121, 722–730. 10.1161/CIRCRESAHA.117.309711 28912179

[B49] Memczak S. Jens M. Elefsinioti A. Torti F. Krueger J. Rybak A.. (2013). Circular RNAs are a large class of animal RNAs with regulatory potency. Nature 495, 333–338. 10.1038/nature11928 23446348

[B50] Methawasin M. Hutchinson K. R. Lee E.-J. Smith J. E. Saripalli C. Hidalgo C. G.. (2014). Experimentally increasing titin compliance in a novel mouse model attenuates the frank-starling mechanism but has a beneficial effect on diastole. Circulation 129, 1924–1936. 10.1161/CIRCULATIONAHA.113.005610 24599837 PMC4032222

[B51] Methawasin M. Strom J. G. Slater R. E. Fernandez V. Saripalli C. Granzier H. (2016). Experimentally increasing the compliance of titin through RNA binding Motif-20 (RBM20) inhibition improves diastolic function in a mouse model of heart failure with preserved ejection fractionclinical perspective. Circulation 134, 1085–1099. 10.1161/CIRCULATIONAHA.116.023003 27630136 PMC5069184

[B52] Millat G. Bouvagnet P. Chevalier P. Sebbag L. Dulac A. Dauphin C.. (2011). Clinical and mutational spectrum in a cohort of 105 unrelated patients with dilated cardiomyopathy. Eur. J. Med. Genet. 54, e570–e575. 10.1016/j.ejmg.2011.07.005 21846512

[B53] Murayama R. Kimura M. Togo-ohno M. Yamasaki,-Y. (2018). Phosphorylation of the RSRSP stretch is critical for splicing regulation by RNA-Binding Motif Protein 20 (RBM20) through nuclear localization. Sci. Rep. 8:8970. 10.1038/s41598-018-26624-w 29895960 PMC5997748

[B54] Nagueh S. F. Shah G. Wu Y. Torre-Amione G. King N. M. P. Lahmers S.. (2004). Altered titin expression, myocardial stiffness, and left ventricular function in patients with dilated cardiomyopathy. Circulation 110, 155–162. 10.1161/01.CIR.0000135591.37759.AF 15238456

[B55] Neagoe C. Opitz C. A. Makarenko I. Linke W. A. (2003). Gigantic variety: expression patterns of titin isoforms in striated muscles and consequences for myofibrillar passive stiffness. J. Muscle Res. Cell Motil. 24, 175–189. 10.1023/A:1026053530766 14609029

[B56] Nielsen J. B. Thorolfsdottir RB Fritsche LG Zhou W Skov MW Graham SE. (2018). Biobank-driven genomic discovery yields new insight into atrial fibrillation biology. Nat. Genet. 50, 1234–1239. 10.1038/s41588-018-0171-3 30061737 PMC6530775

[B57] Opitz C. A. Leake M. C. Makarenko I. Benes V. Linke W. A. (2004). Developmentally regulated switching of titin size alters myofibrillar stiffness in the perinatal heart. Circ. Res. 94, 967–975. 10.1161/01.RES.0000124301.48193.E1 14988228

[B58] Opitz C. A. Linke W. A. (2005). Plasticity of cardiac titin/connectin in heart development. J. Muscle Res. Cell Motil. 26, 333–342. 10.1007/s10974-005-9040-7 16465471

[B59] Pérez-Serra A. Toro R. Sarquella-Brugada G. De Gonzalo-Calvo D. Cesar S. Carro E.. (2016). Genetic basis of dilated cardiomyopathy. Int. J. Cardiol. 224, 461–472. 10.1016/j.ijcard.2016.09.068 27736720

[B60] Radke M. H. Peng J. Wu Y. McNabb M. Nelson O. L. Granzier H.. (2007). Targeted deletion of titin N2B region leads to diastolic dysfunction and cardiac atrophy. Proc. Natl. Acad. Sci. U.S.A. 104, 3444–3449. 10.1073/pnas.0608543104 17360664 PMC1805563

[B61] Rampersaud E. Siegfried J. D. Norton N. Li D. Martin E. Hershberger R. E. (2011). Rare variant mutations identified in pediatric patients with dilated cardiomyopathy. Prog. Pediatr. Cardiol. 31, 39–47. 10.1016/j.ppedcard.2010.11.008 21483645 PMC3072577

[B62] Refaat M. M. Lubitz S. A. Makino S. Islam Z. Michael Frangiskakis J. Mehdi H.. (2012). Genetic variation in the alternative splicing regulator RBM20 is associated with dilated cardiomyopathy. HRTHM 9, 390–396. 10.1016/j.hrthm.2011.10.016 22004663 PMC3516872

[B63] Rivas-Pardo J. A. Eckels E. C. Popa I. Kosuri P. Linke W. A. Fernández J. M. (2016). Work done by titin protein folding assists muscle contraction. Cell Rep. 14, 1339–1347. 10.1016/j.celrep.2016.01.025 26854230 PMC4865255

[B64] Sabater-Molina M. Pérez-Sánchez I. Hernández del Rincón J. P. Gimeno J. R. (2018). Genetics of hypertrophic cardiomyopathy: a review of current state. Clin. Genet. 93, 3–14. 10.1111/cge.13027 28369730

[B65] Schweingruber C. Rufener S. C. Zünd D. Yamashita A. Mühlemann O. (2013). Nonsense-mediated mRNA decay — mechanisms of substrate mRNA recognition and degradation in mammalian cells. Biochim. Biophys. Acta Gene Regul. Mech. 1829, 612–623. 10.1016/j.bbagrm.2013.02.005 23435113

[B66] Streckfuss-Bömeke K. Tiburcy M. Fomin A. Luo X. Li W. Fischer C.. (2017). Severe DCM phenotype of patient harboring RBM20 mutation S635A can be modeled by patient-specific induced pluripotent stem cell-derived cardiomyocytes. J. Mol. Cell. Cardiol. 113, 9–21. 10.1016/j.yjmcc.2017.09.008 28941705

[B67] van den Hoogenhof M. M. G. Beqqali A. Amin A. S. van der Made I. Aufiero S. Khan M. A. F.. (2018). RBM20 mutations induce an arrhythmogenic dilated cardiomyopathy related to disturbed calcium handling. Circulation 138, 1330–1342. 10.1161/CIRCULATIONAHA.117.031947 29650543

[B68] Wang K. McClure J. Tu A. (1979). Titin: major myofibrillar components of striated muscle. Proc. Natl. Acad. Sci. U.S.A. 76, 3698–3702. 10.1073/pnas.76.8.3698 291034 PMC383900

[B69] Warren C. M. Jordan M. C. Roos K. P. Krzesinski P. R. Greaser M. L. (2003). Titin isoform expression in normal and hypertensive myocardium. Cardiovasc. Res. 59, 86–94. 10.1016/S0008-6363(03)00328-6 12829179

[B70] Warren C. M. Krzesinski P. R. Campbell K. S. Moss R. L. Greaser M. L. (2004). Titin isoform changes in rat myocardium during development. Mech. Dev. 121, 1301–1312 10.1016/j.mod.2004.07.003 15454261

[B71] Wells Q. S. Becker J. R. Su Y. R. Mosley J. D. Weeke P. D'Aoust L.. (2013). Whole exome sequencing identifies a causal RBM20 mutation in a large pedigree with familial dilated cardiomyopathy. Circ. Cardiovasc. Genet. 6, 317–326. 10.1161/CIRCGENETICS.113.000011 23861363 PMC3895490

[B72] Wyles S. Hrstka S. Reyes S. Terzic A. Olson T. Nelson T. (2016a). Pharmacological modulation of calcium homeostasis in familial dilated cardiomyopathy: an *in vitro* analysis from an RBM20 patient-derived iPSC model. Clin. Transl. Sci. 9, 158–167. 10.1111/cts.12393 27105042 PMC4902766

[B73] Wyles S. P. Li X. Hrstka S. C. Reyes S. Oommen S. Beraldi R.. (2016b). Modeling structural and functional deficiencies of *RBM20* familial dilated cardiomyopathy using human induced pluripotent stem cells. Hum. Mol. Genet. 25, 254–265. 10.1093/hmg/ddv468 26604136 PMC4706113

[B74] Xiao S. H. Manley J. L. (1997). Phosphorylation of the ASF/SF2 RS domain affects both protein-protein and protein-RNA interactions and is necessary for splicing. Genes Dev. 11, 334–344. 10.1101/gad.11.3.334 9030686

[B75] Yeakley J. M. Tronchère H. Olesen J. Dyck J. A. Wang H. Y. Fu X. D. (1999). Phosphorylation regulates *in vivo* interaction and molecular targeting of serine/arginine-rich pre-mRNA splicing factors. J. Cell Biol. 145, 447–455. 10.1083/jcb.145.3.447 10225947 PMC2185075

[B76] Zahler A. M. Lane W. S. Stolk J. A. Roth M. B. (1992). SR proteins: a conserved family of pre-mRNA splicing factors. Genes Dev. 6, 837–847. 10.1101/gad.6.5.837 1577277

[B77] Zahr H. C. Jaalouk D. E. (2018). Exploring the crosstalk between LMNA and splicing machinery gene mutations in dilated cardiomyopathy. Front. Genet. 9:231. 10.3389/fgene.2018.00231 30050558 PMC6052891

[B78] Zerbino D. R. Achuthan P. Akanni W. Amode M. R. Barrell D. Bhai J.. (2018). Ensembl 2018. Nucleic Acids Res. 46, D754–D761. 10.1093/nar/gkx1098 29155950 PMC5753206

[B79] Zhao Y. Feng Y. Zhang Y.-M. Ding X.-X. Song Y.-Z. Zhang A.-M.. (2015). Targeted next-generation sequencing of candidate genes reveals novel mutations in patients with dilated cardiomyopathy. Int. J. Mol. Med. 36, 1479–1486. 10.3892/ijmm.2015.2361 26458567 PMC4678153

[B80] Zhu C. Guo W. (2017). Detection and quantification of the giant protein titin by SDS-agarose gel electrophoresis. MethodsX 4, 320–327. 10.1016/j.mex.2017.09.007 29872636 PMC5986978

[B81] Zhu C. Yin Z. Ren J. McCormick R. J. Ford S. P. Guo W. (2015). RBM20 is an essential factor for thyroid hormone-regulated titin isoform transition. J. Mol. Cell Biol. 7, 88–90. 10.1093/jmcb/mjv002 25573899 PMC4400401

[B82] Zhu C. Yin Z. Tan B. Guo W. (2017). Insulin regulates titin pre-mRNA splicing through the PI3K-Akt-mTOR kinase axis in a RBM20-dependent manner. Biochim. Biophys. Acta Mol. Basis Dis. 1863, 2363–2371. 10.1016/j.bbadis.2017.06.023 28676430 PMC5547897

